# AQUARIUM_HB: a bioinformatics pipeline for human blood circular RNA analysis

**DOI:** 10.1016/j.ncrna.2025.09.004

**Published:** 2025-09-15

**Authors:** Shaoxun Yuan, Xue Bai, Linwei Li, Wanjun Gu

**Affiliations:** a School of Artificial Intelligence and Information Technology, Nanjing University of Chinese Medicine, Nanjing, Jiangsu, 210023, China; b Jiangsu Province Engineering Research Center of TCM Intelligence Health Service, Nanjing University of Chinese Medicine, Nanjing, Jiangsu, 210023, China; c Collaborative Innovation Center of Jiangsu Province of Cancer Prevention and Treatment of Chinese Medicine, Nanjing University of Chinese Medicine, Nanjing, Jiangsu, 210023, China

**Keywords:** AQUARIUM-HB, circular RNA, bioinformatics, blood, RNA-seq

## Abstract

Accurately identifying and quantifying human blood circular RNAs (circRNAs) from RNA-seq data is a critical bioinformatics challenge in biomarker discovery for human diseases. In this study, we present *AQUARIUM-HB*, a comprehensive bioinformatics pipeline for identifying, quantifying, annotating, and analyzing circRNAs from human blood transcriptomes. *AQUARIUM-HB* includes three functional modules. First, it identifies and annotates circRNAs from rRNA-depleted RNA-seq datasets of human blood samples. Second, it performs an in-depth expression analysis of blood circRNAs. Third, it constructs a reference set of full-length blood circRNAs. We demonstrate the application of *AQUARIUM-HB* using a human blood RNA-seq dataset from COVID-19 patients, showcasing its potential for improving the accuracy and depth of circRNA biomarker discovery.

## Highlights

• *AQUARIUM-HB* identifies and quantifies circRNAs from blood HT RNA-seq data at transcript level.
• *AQUARIUM-HB* dynamically builds a reference of blood circRNAs, enhancing identification and quantification accuracy.
• *AQUARIUM-HB* can be applied to discover circRNA biomarkers from blood HT RNA-seq data for human diseases.

## 1 Introduction

Liquid biopsies, which use body fluids such as blood or urine, provide non-invasive, real-time approach for disease monitoring compared to traditional tissue biopsies [1]. Peripheral blood is particularly favored for its ease of collection, minimal invasiveness and comprehensive information content. Among the numerous biomarkers found in blood, including circulating tumor cells and extracellular vesicles, RNA-based molecular markers have gained significant attention due to their dynamic expression patterns and close association with disease states [2,3]. Circular RNAs (CircRNAs), in particular, stand out because of their higher stability [4] and specificity [5] compared to traditional linear RNAs. Recent studies have demonstrated crucial roles of blood circRNAs in intercellular communication and disease progression of severe diseases such as cancers [6], suggesting their promising application in liquid biopsies [7].

The identification and quantification of circRNAs from high-throughput RNA sequencing (HT RNA-seq) of rRNA-depleted blood samples allow for a more profound understanding of circRNA expression dynamics. This understanding enhances their potential utility in diagnosing and prognosing complex diseases. Although various computational tools with differing performance levels have been developed for circRNA quantification from HT RNA-seq data [[8], [9], [10], [11], [12]], several challenges remain [13]. For example, most existing tools quantify circRNA expression by normalizing read counts across back-splice junction (BSJ) sites [8,[10], [11], [12],^14^]. However, this approach may introduce biased estimation of circRNA expression due to the uneven coverage of sequencing reads and the generally lower expression levels of circular transcripts. In our previous studies, we proposed a pseudo-linear transformation for circular transcripts [15] and implemented a model-based strategy to quantify circRNAs using the full-length RNA structure of these transcripts [16]. This computational framework has been shown to accurately and simultaneously quantify the expression levels of both circular and linear RNA transcripts from rRNA-depleted HT RNA-Seq data [4,6]. In particular, the full-length structure of circRNAs in peripheral blood samples can enhance the accuracy of circRNA quantification [16]. Therefore, establishing a reference set of human blood full-length circRNAs is crucial for accurate circRNA quantification from RNA-seq datasets.

Full-length circRNAs can be obtained from two sources. First, Oxford Nanopore Technology (ONT) has been successfully used to identify full-length circRNAs from tissue samples and cell lines of several model organisms, including *circFL-seq* [17], *circNick-LRS* [18], *IsoCirc* [19] and *CIRI-long* [20]. For instance, Xin et al. identified 107,147 full-length circRNA isoforms across 12 human tissues and one human cell line, which are publicly accessible through the UCSC Genome Browser [19]. Additionally, two public circRNA databases, *FLcircAS* [21] and *circAtlas* [22,23], have compiled over one million human full-length circRNA isoforms from ONT datasets. Second, several computational tools have been developed to reconstruct the internal structure of circular transcripts from HT RNA-seq data [14]. For example, *CIRI-full* utilizes BSJ sites detected from *CIRI2* and reverse overlap features of short sequencing reads to assemble full-length circRNA sequences [14], thus enabling the distinction of alternatively spliced circular RNA isoforms. Other tools, such as *psirc* [24], c*ircRNA-full* [25], *CYCLER* [26] and *FEICP* [27], leverage chimeric alignment information from short sequencing reads to reconstruct full-length circular transcripts. Given the wealth of publicly available HT RNA-seq datasets, this second source remains the primary means of assessing genome-wide full-length circRNA profiles.

**Fig. 1:**
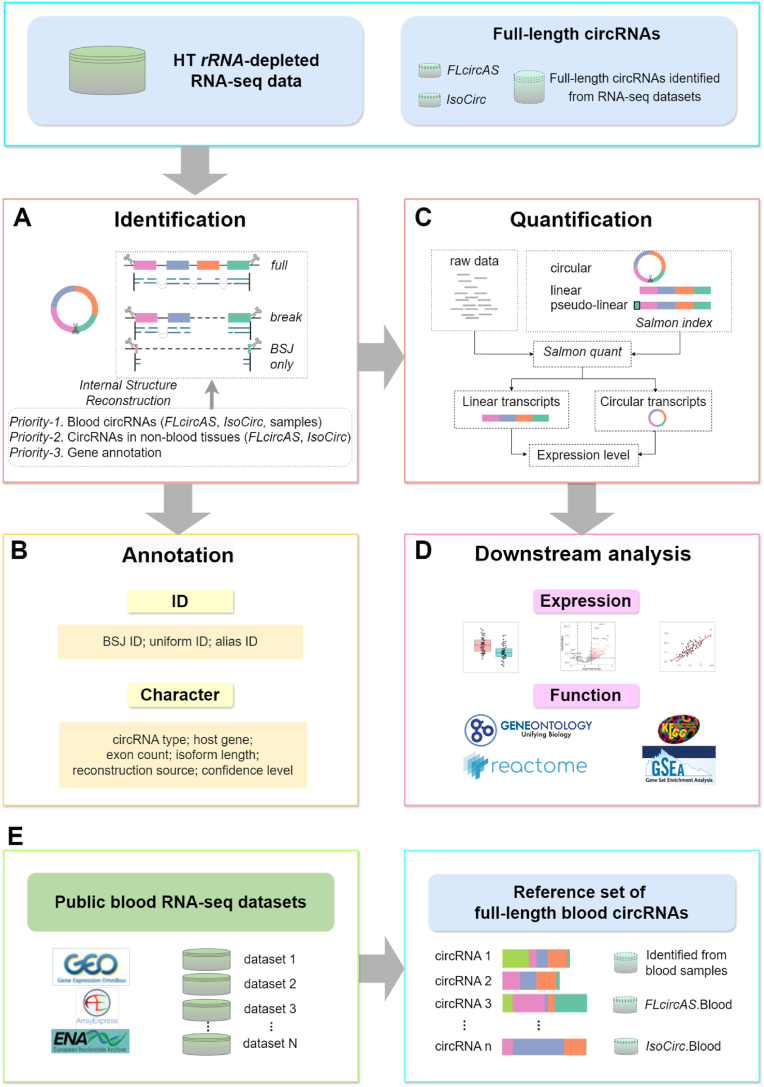
**The pipeline of *AQUARIUM-HB*. (A) Identification of blood circRNA.** For circRNAs that are identified as the “*full*” transcripts by *CIRI-full* [14], the complete sequences from the *CIRI-full* [14] output were used in the subsequent analysis. For circRNAs that were identified as the “*break*” or “*BSJ only*” transcripts by *CIRI-full* [14], blood full-length circRNAs from the *FLcircAS* [21] and *IsoCirc* [19] databases, along with “*full*” transcripts obtained from blood samples, were prioritized for reconstruction. Alternative, full-length circRNAs from non-blood in the *FLcircAS* [21] and *IsoCirc* [19] databases were utilized. Gene annotation of the human genome was least preferred for reconstruction. **(B) Annotation of blood circRNAs.** The annotation consists of two parts: *ID* and *character*. The ID includes the BSJ position of a circRNA, the standardized circRNA nomenclature [28], and the alias names corresponding to various circRNA databases [21,23,29,30]. The character includes various attributes of blood circRNAs, including the types of circRNAs (exon, intron, and intergenic), the corresponding host genes, exon details associated with the circRNAs, their presence in *FLcircAS* [21] or *IsoCirc* [19] databases, and the confidence levels regarding the identification of these circRNAs. (**C) Quantification of blood circRNAs.** The expressions of both linear and circular transcripts were quantified simultaneously at the transcript per million (TPM) level using *sailfish-cir* [15]. **(D) Downstream analysis of blood circRNAs.** Expression analysis involves differential analysis of circRNAs across groups and comparison with the expression of linear RNAs. Enrichment analysis includes functional analysis of circRNAs using *GO* [34], *KEGG* [35], *Reactome* [36], and *GSEA* [37]. **(E) Reference set update of full-length blood circRNAs.** The reference set of full-length blood circRNAs includes two components. The first component integrates the full-length blood circRNAs deposited in the *FLcircAS* [21] and *IsoCirc* [19] databases. The second component includes the full-length circRNAs identified from public HT RNA-seq datasets of human blood samples. The second component is dynamic, allowing continuous expansion and updates as new HT RNA-seq datasets become available.

To overcome the need for reliable full-length reconstruction of human blood circRNAs while simultaneously quantifying and analyzing both linear and circular transcripts from HT RNA-seq data, we developed *AQUARIUM-HB*. This pipeline identifies, annotates, quantifies, and performs expression analysis of human blood circRNAs from HT RNA-seq datasets (Fig. 1). Using this pipeline, the reference set of full-length blood circRNAs can be dynamically expanded by incorporating additional human blood HT RNA-seq datasets. By incorporating known full-length circRNAs from public databases and dynamically expanding its reference set with new RNA-seq datasets, *AQUARIUM-HB* aims to improve the identification and quantification of blood circRNAs, thus advancing their application in biomarker discovery and contributing to improved diagnostic and therapeutic strategies.

## 2 Methods

### 2.1 Identification of human blood circRNAs from HT RNA-seq data

In *AQUARIUM* [16], we incorporated the reconstructed circRNAs from *CIRI-full* [14] to improve the quantification accuracy of circRNA expressions. Due to the short length of sequencing reads or insufficient sequencing depth of HT RNA-seq, *CIRI-full* may not completely characterize the full-length sequence of some circRNAs. In this case, *AQUARIUM* utilizes the gene annotation of human genome to facilitate the internal structure reconstruction of circRNAs [16]. However, this strategy does not account the internal structures in large number of known full-length circRNAs derived from ONT or HT RNA-seq datasets. To increase the accuracy of circRNA quantification, we integrated the full-length circRNAs in *FLcircAS* [21] or *IsoCirc* [19] databases in the *AQUARIUM-HB* pipeline, together with the full-length circRNAs obtained from *CIRI-full* identification of HT RNA-seq datasets (Fig. 1A). We reconstructed the full-length sequence of circRNAs identified by *CIRI-full* as follows. For circRNAs that are identified as the “*full*” transcripts by *CIRI-full*, the complete sequences from the *CIRI-full* output were used in subsequent analysis. For circRNAs that were identified as the “*break*” or “*BSJ only*” transcripts by *CIRI-full*, the internal structures of human full-length circRNAs from different sources were used to reconstruct the full-length sequence of these incomplete circRNAs. First, blood full-length circRNAs in the *FLcircAS* [21] or *IsoCirc* [19] databases, along with those full-length transcripts identified from blood HT RNA-seq datasets, were prioritized for internal structure reconstruction (*Priority-1*). If this is insufficient, circRNAs from non-blood tissues in the *FLcircAS* [21] or *IsoCirc* [19] databases were utilized (*Priority-2*). Lastly, if required, gene annotations are employed to complete circRNA structures, ensuring robust and comprehensive identification (*Priority-3*).

### 2.2 Annotation of human blood circRNAs

The identified human blood circRNAs were annotated using the terms listed in Table 1 (Fig. 1B). First, each circRNA is assigned a *uniform ID* with the terminology proposed by Chen et al. [28], ensuring its consistency across different databases. For example, *circUBXN4(2,3,L4,5)*, which *2,3,4,5* indicate exons 2, 3, 4 and 5, while *L* before exon 4 indicate 5′ alternative splicing of exon 4. To connect with the knowledge deposited in other circRNA databases, the *aliases ID* of each circRNA in several existing circRNA databases, including *FLcircAS* [21], *TransCirc* [29], *circAtlas* [22,23], *circBase* [30], and *PltDB* [31], were annotated as well. Next, the *reconstruction source* of each circRNA is documented, indicating whether it is reconstructed by *CIRI-full* from RNA-seq data, or it is complemented by full-length circRNAs in *FLcircAS* and/or *IsoCirc* databases. Finally, circRNAs are classified by *confidence level* based on their reconstruction method and detection frequency. The *Level-1* circRNAs should meet two criteria. First, they should have their full-length sequences reconstructed by *CIRI-full*. Second, these circRNAs are detected in at least five samples by *CIRI-full*, or they have been deposited in the *FLcircAS* and/or *IsoCirc* databases. The *Level-2* circRNAs should have their full-length sequences reconstructed by *CIRI-full*, be detected in less than five samples, and not be deposited in the *FLcircAS* and/or *IsoCirc* databases. The *Level-3* circRNAs are those incomplete circRNAs that are reconstructed using full-length circRNAs from *FLcircAS* and/or *IsoCirc* and/or HT RNA-seq blood samples. The *Level-4* circRNAs are those incomplete circRNAs that are reconstructed using gene annotation of human genome.

**Table 1:** The terms used in circRNA annotation module of *AQUARIUM-HB* pipeline.

Annotation term	Description
***BSJ ID***	BSJ position of a circRNA
***uniform ID***	standard nomenclature of circRNA (Chen et al. [28])
***alias ID***	aliases ID in several circRNA databases [[21], [22], [23],[29], [30], [31]]
***circRNA type***	type of a circRNA (exonic, intronic or intergenic)
***host gene***	host gene ID(s) of an exonic or intronic circRNA
***exon count***	the number of exons in a circRNA
***sequence length***	the splicing length of a circRNA
***reconstruction source***	reconstruction source of a circRNA
***confidence level***	the circRNA confidence level according to the strategies used in identification and reconstruction

### 2.3 Expression analysis of human blood circRNAs

Following reconstruction, we quantified the expression of both linear and circular transcripts simultaneously at the transcript level using *AQUARIUM* [16], a model-based framework that we have developed for circRNA quantification (Fig. 1C). To minimize the interference from highly abundant transcripts in blood samples, we ignored the expression of both circular and linear RNA transcripts from 13 hemoglobin related gene and 171 ribosomal genes in *HGNC* database [32]. Next, we kept only the transcripts from protein-coding genes and recalculated the TPM (Transcripts per Million) expression values for all circular and linear transcripts. For each circRNA, TPM values at the isoform, BSJ, and gene levels were aggregated, providing a comprehensive profile of circRNA expressions. Differential expression analyses across groups were performed using *DESeq2* [33], with subsequent gene set enrichment analyses in *GO* [34], *KEGG* [35], *Reactome* [36], and *GSEA* [37] to reveal underlying biological functions and pathways (Fig. 1D).

### 2.4 Dynamic expansion of the reference set of human blood full-length circRNAs

A high-quality reference set of human blood full-length circRNAs can improve the accuracy of circRNA identification and quantification in peripheral blood samples. *AQUARIUM-HB* is capable of constructing a reference set of human blood full-length circRNAs sourced from the ONT and HT RNA-seq datasets (Fig. 1E). This reference set includes two components. The first is derived from full-length circRNAs in the *FLcircAS* [21] and *IsoCirc* [19] databases. The second component includes the full-length circRNAs identified from public HT RNA-seq datasets of human blood samples. Notably, the second component is dynamic, allowing continuous expansion and updates as new HT RNA-seq datasets become available. This iterative enhancement ensures both accuracy and comprehensiveness in circRNA identification and quantification from human blood samples, advancing our understanding of their roles in diseases.

### 2.5 Data

To exemplify our *AQUARIUM-HB* pipeline, we downloaded a HT RNA-seq dataset from the GEO database [38] (accession number: GSE172114). This dataset includes 69 whole blood RNA samples from COVID-19 patients, comprising 46 critical and 23 non-critical patients at the time of hospitalization [39]. RNA-seq libraries were prepared using the *TruSeq* Stranded Total RNA with *Ribo-Zero* Globin kit (Illumina) and sequenced on the *Illumina NovaSeq* 6000 platform with S4 flow cells, generating 151-base pair paired-end reads.

### 2.6 Comparison of identification and quantification performance of different tools

To evaluate the potential quantification bias introduced by reference-based adjustment, a comparative analysis using circRNAs identified by different tools (*CIRIquant*, *CircExplorer*, *AQUARIUM*, and *AQUARIUM*-*HB*) were performed from PRJNA722046.

We simulated bulk RNA-seq data using the *simulate_experiment_countmat* function from the “*polyester*” package [40] (version 1.34.0) in R (version 4.2.0), based on the *AQUARIUM-HB* reference set derived from PRJNA722046 (comprising 331,544 full-length isoforms and 115,392 BSJs). The linear and circular transcript sequences and their expression abundances from sample SAMN18743932 in PRJNA722046 served as templates for simulation.

First, we ran *AQUARIUM-HB* on the PRJNA722046 dataset, obtaining expression values for 3182 circRNAs and 101,951 linear RNAs in sample SAMN18743932. To simulate circRNA transcripts, each circRNA sequence was duplicated 10 times to construct pseudo BSJ sites. The “*readmat*” and “*len*” parameters of *simulate_experiment_countmat* were set based on the transcript expression values and expected sequencing read lengths. Four simulated RNA-seq datasets were generated, all with pair-end sequencing and four read lengths: 75 bp, 100 bp, 150 bp, and 200 bp. For each read length, we also included three biological replicates. Subsequently, the results for each read length were averaged across the three replicates.

*AQUARIUM-HB*, *AQUARIUM*, *CIRIquant* (version 1.1.3), and *CIRCexplorer* (version 2.3.8) were applied to four simulate datasets for comprehensive evaluation in two aspects: 1) the overlapping of isoform-level or BSJ-level circRNAs identified by each method compared to the sample SAMN18743932; 2) the correlation between identified isoform/BSJ-level circRNA expressions and the true expression levels in SAMN18743932.

## 3 Results

### 3.1 Identification of human blood circRNAs

**Fig. 2:**
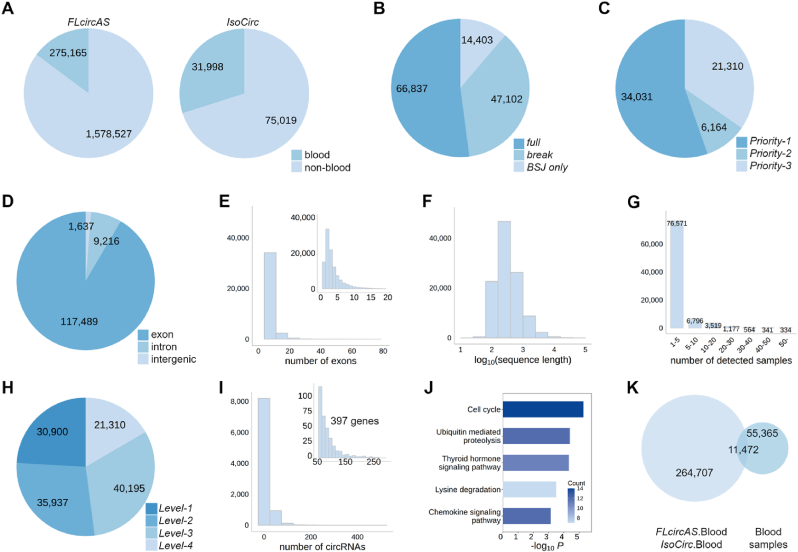
**Characteristics of circRNAs in blood. (A)** Proportional distribution of blood and non-blood circRNAs in the *FLcircAS* [21] and *IsoCirc* [19] databases. **(B)** Distribution of “*full*”, “*break*” and “*BSJ only*” circRNAs in select dataset. **(C)** Priority distribution during the reconstruction pipeline of “*break*” and “*BSJ only*” circRNAs. *Priority-1* indicates incomplete circRNAs are reconstructed using blood full-length circRNAs in the *FLcircAS* [21] or *IsoCirc* [19] databases, along with those full-length transcripts identified from blood RNA-seq datasets. *Priority-2* indicates incomplete circRNAs are reconstructed using non-blood tissues in the *FLcircAS* [21] or *IsoCirc* [19] databases. *Priority-3* indicates using gene annotation of human genome for reconstruction. **(D)** Distribution of identified circRNA types according to positions of its two ends on chromosome. **(E)** Histogram of the exon count distribution for identified circRNAs. **(F)** Histogram of the isoform length distribution of identified circRNAs. **(G)** Detected sample of identified circRNAs in the select dataset. **(H)** Proportional distribution of confidence levels for identified circRNAs. **(I)** Histogram of gene-circular isoform count distribution. **(J)** KEGG pathway enrichment results for genes with highly variable splicing levels (corresponding to more than 50 transcripts). **(K)** The reference set of full-length blood circRNAs includes two parts: full-length blood circRNAs in the *FLcircAS* [21] and *IsoCirc* [19] databases, and full-length circRNAs identified from the selected HT RNA-seq dataset.

We first applied the *AQUARIUM-HB* pipeline to analyze HT RNA-seq data from whole blood samples of 69 COVID-19 patients [39]. Using databases *FLcircAS* [21] and *IsoCirc* [19], we retrieved 275,165 and 31,998 blood-derived circRNAs, respectively. Among these, *FLcircAS* contains 275,165 blood-derived circRNAs (14.8 %), and *IsoCirc* contains 31,998 blood-derived circRNAs (29.9 %) (Fig. 2A). Next, the internal structures of circRNAs from the HT RNA-seq data were reconstructed by *CIRI-full* using the identified BSJ sites and overlapping sequences between paired-end reads. In this dataset, a total of 128,342 circRNAs were identified. Among 128,342 circRNAs, the number of overlapping circRNAs with those recorded in the *circAtlas* database is 15,285; and the number of overlapping ones with those in the *circBase* database is 9536 (Figure S1). Of these, the full-length sequences of 66,837 (52.1 %) circRNAs were completely reconstructed, while the remaining circRNAs were partially reconstructed (47,102 circRNAs, 36.7 %) or only had the BSJ site identified (14,403 circRNAs, 11.2 %) (Fig. 2B). Then, these partially reconstructed circRNAs or BSJ only circRNAs were supplementally extended using the pipeline's strategy (Fig. 1A). Among them, 34,031 circRNAs (55.3 %) were supplemented using existing blood-derived full-length circRNAs in databases or from blood samples (*Priority-1*, Fig. 2C). 6164 circRNAs (10 %) were completed by full-length circRNAs from non-blood tissues in *FLcircAS* [21] or *IsoCirc* [19] databases (*Priority-2*, Fig. 2C). The remaining 21,310 circRNAs (34.6 %) were supplemented using gene annotation of human genome (*Priority-3*, Fig. 2C). These results indicate that the majority of partially reconstructed circRNAs could be effectively supplemented using known full-length circRNAs in blood, underscoring the importance of using blood full-length circRNA databases in reconstruction of circRNA internal structure. Additionally, the use of known full-length circRNAs in non-blood tissues and the genomic annotation can facilitate the pipeline's ability in addressing the gaps in circRNA internal structure.

### 3.2 Annotation of human blood circRNAs

Next, these 128,342 blood circRNAs were annotated by our *AQUARIUM-HB* pipeline (Fig. 1B). Among them, a majority (117,489, 91.5 %) are exonic circRNAs (Fig. 2D) with five or five less exons (Fig. 2E). In terms of transcript length, most circRNAs are shorter than 1000 base pairs (Fig. 2F). Regarding the confidence level, most circRNAs (76,571, 85.7 %) were identified in less than five samples (Fig. 2G), indicating potential sample specificity or low expression levels. For 52.1 % circRNAs that are completely reconstructed by *CIRI-full*, almost half (30,900, 46.2 %) were identified in at least five samples or already deposited in *FLcircAS* and/or *IsoCirc* databases (*Level-1*, Fig. 2H). The remaining 35,937 *CIRI-full* completely reconstructed blood circRNAs were newly identified in human blood (*Level-2*, Fig. 2H). For 47.9 % circRNAs that are incompletely reconstructed by *CIRI-full*, 40,195 (65.4 %) were complementally supplemented by full-length circRNAs in *FLcircAS* or *IsoCirc* databases or blood samples (*Level-3*, Fig. 2H), while the remaining 21,310 (34.6 %) were completed using gene annotation of human genome (*Level-4*, Fig. 2H). These blood circRNAs were associated with 9308 human genes, with most genes transcribed only a single circRNA (Fig. 2I). However, 397 genes exhibited high levels of alternative splicing of circular transcripts, with each of these genes corresponding to more than 50 transcripts. Functional enrichment analysis showed these highly spliced genes are significantly involved in pathways like cell cycle regulation, ubiquitin-mediated proteolysis, and chemokine signaling (Fig. 2J).

### 3.3 Expression analysis of circRNA profiles

**Fig. 3:**
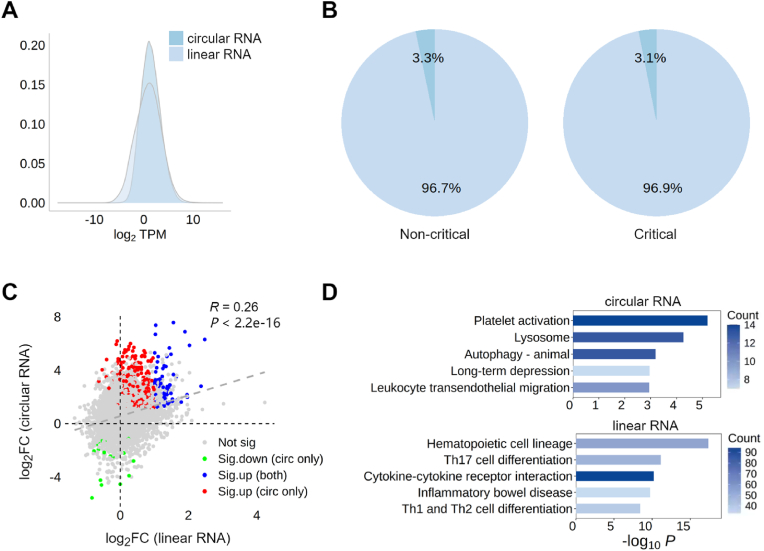
**Expression profiling analysis of circRNA and linear RNA. (A)** Density plot of the expression distributions of both circular and linear transcripts. **(B)** Proportional expression of circRNAs and linear RNAs in the non-critical and critical COVID-19 patients. **(C)** The correlation of log_2_(fold change) of circRNAs versus log_2_(fold change) of corresponding linear RNA expression. Blue dots represent transcription-derived circRNAs that were up-regulated because of consistent up-regulation of their parent genes. Red and green dots represent splice-derived circRNAs that were up-regulated or down-regulated whose parent genes showed no significant expression changes. Grey dots represent circRNAs that had no differential expression. **(D)** The top 5 KEGG functional enrichment plots of differentially expressed circRNAs and linear RNAs.

CircRNAs generally exhibit lower expression levels than their linear counterparts. can exhibit varying expression levels from the same gene depending on the context [41,42]. Understanding the regulation of dynamic circRNA expression highlights the importance of simultaneously quantifying both circular and linear RNA types. Using the *AQUARIUM-HB* pipeline, we generated a density plot of RNA expression of both circular and linear transcripts to illustrate the expression distribution of circRNAs and linear mRNAs (Fig. 3A). The overall expression of circRNAs and linear RNAs follows a normal distribution, indicating that their expression levels are well-regulated and may reflect typical biological variability across samples. The expression levels of circRNAs much smaller than linear mRNA transcripts in both non-critical and critical COVID-19 patients (Fig. 3B). The circRNAs accounted for 3.3 % of total RNA expression in non-critical COVID-19 patients, slightly higher than that in critical patients (3.1 %). Furthermore, a significant positive correlation was observed between the expression changes of circRNAs and their corresponding linear RNAs at the gene level (*R* = 0.26, *P-value* < 2.2∗10^−16^) (Fig. 3C). This suggests the expressional change of circRNAs are largely determined by the transcriptional regulation of its host gene. For example, expressions of some circRNAs are up-regulated (Fig. 3C, blue dots) due to the up-regulation of their corresponding parent gene. However, some dysregulated circRNAs are splicing-derived circRNAs, with their expression levels up-regulated (Fig. 3C, red dots) or down-regulated (Fig. 3C, green dots) independently to the transcriptional regulation of their parent genes. The functional enrichment patterns of differentially expressed circRNAs also differ from those of differentially expressed linear RNAs, indicating distinct biological roles of circRNAs in disease severity of COVID-19 (Fig. 3D).

### 3.4 Construction of a reference set of human blood full-length circRNAs

Although long-read sequencing technology can directly sequence the full-length circRNAs, it still has limitations in application, such as high costs. On the other hand, the whole genome characterization of circRNAs can be achieved through the HT RNA-seq technology with various RNA enrichment strategies. Given the importance of a reliable reference set, *AQUARIUM-HB* dynamically integrates circRNAs from both *FLcircAS* [21] and *IsoCirc* [19] databases, along with newly identified circRNAs from HT RNA-seq datasets. Initially, 275,165 blood full-length circRNAs from the *FLcircAS* database [21] and 31,998 blood full-length circRNAs from the *IsoCirc* database [19] were integrated as the reference set of human blood full-length circRNAs (Supplementary Table S1). This initial reference set was composed of 276,179 blood-derived full-length circRNAs from existing circRNA databases in total. Our pipeline identified 66,837 full-length circRNAs from the HT RNA-seq data of 69 human blood samples of COVID-19 patients. This set of full-length circRNAs in human blood samples were then used to update the initial reference set (Fig. 1E). Among them, 11,472 full-length circRNAs were already deposited in the *FLcircAS* or *IsoCirc* databases, while the remaining 55,365 circRNAs were newly identified in human blood samples (Fig. 2K). Finally, we obtained an updated human full-length blood circRNA reference set consisting of 331,544 full-length circRNAs. This reference set provides a robust foundation for circRNA identification and quantification in blood, supporting advanced research in biomarker discovery and improving diagnostic accuracy.

### 3.5 Results of identification and quantification performance of different tools

**Fig. 4:**
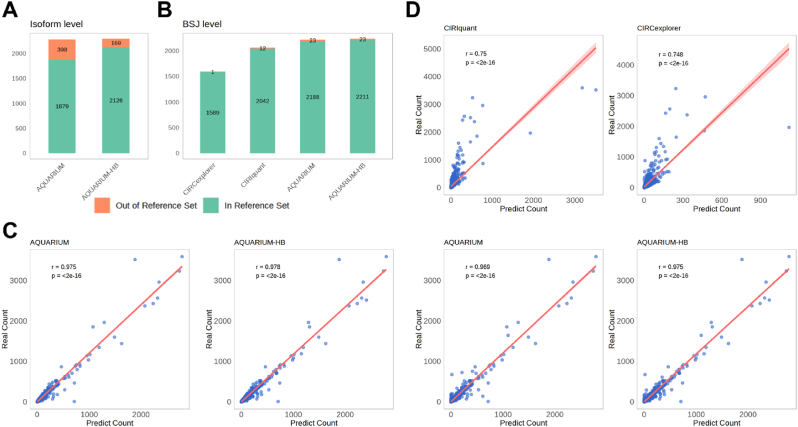
Comparisons among *CircExplorer* [10]*, CIRIquant* [3], *AQUARIUM* [11] and *AQUARIUM*-*HB* (Read Length 150). Numbers of circRNA in reference set and out of reference set at isoform **(A)** and BSJ level **(B)**. Isoform-level circRNA expression: tool-detected values (X-axis) vs. real sample-estimated values (Y-axis). **(C)** BSJ-level circRNA expression: tool-detected values (X-axis) vs. real sample-estimated values (Y-axis). Pearson correlation coefficients (r) and p-values are shown.

At the isoform level, across all sequencing read lengths, *AQUARIUM-HB* identified significantly more circRNAs that overlapped with the true data in SAMN18743932 compared to *AQUARIUM* (Fig. 4A, Supplementary Figure S2A). At the BSJ level, *AQUARIUM-HB* showed comparable performance to *CIRIquant* in overlapping circRNA identification, outperforming *AQUARIUM* slightly and significantly surpassing *CIRCexplorer* (Fig. 4B, Supplementary Figure S2B).

Regarding expression correlation, *AQUARIUM-HB* marginally outperformed *AQUARIUM* at both the isoform and BSJ levels. At the BSJ level, *AQUARIUM-HB* significantly outperformed *CIRIquant* and *CIRCexplorer*, demonstrating stronger consistency with the true expression levels in SAMN18743932 (Fig. 4C and D, Supplementary Figure S2C, Supplementary Figure S2D).

These results collectively highlight *AQUARIUM-HB*'s superiority in circRNA identification reproducibility, isoform reconstruction robustness, and expression correlation, particularly when compared to conventional methods across diverse sequencing read lengths.

## 4 Discussion

In this study, we constructed *AQUARIUM-HB*, a comprehensive pipeline capable of identifying and quantifying circRNAs at the transcript level from HT RNA-seq data of human peripheral blood samples. *AQUARIUM-HB* presents several substantial improvements over the original *AQUARIUM* [16] pipeline. While *AQUARIUM* relied solely on full-length circRNA isoforms reconstructed from short-read RNA-seq data of human samples, *AQUARIUM-HB* integrates high-confidence long-read datasets from *FLcircAS* and *IsoCirc* to enhance isoform reconstruction. Additionally, *AQUARIUM-HB* establishes a comprehensive blood-derived full-length circRNA isoform reference set and provides systematic annotation of isoform structure and function.

We included a systematic comparison between *AQUARIUM-HB* and other established circRNA analysis pipelines (Table S2). Moreover, we conducted a comparative analysis using circRNAs identified by *CIRIquant* [11], *CircExplorer* [43], *AQUARIUM* [16], and *AQUARIUM*-*HB* using a simulate data. These results demonstrate that *AQUARIUM-HB* outperforms existing methods in terms of isoform completeness and annotation quality in blood-derived datasets.

*AQUARIUM-HB* also has several limitations. First, it currently requires HT RNA-seq data to be paired-end with equal read lengths at both ends; though, for datasets with unequal read lengths, preprocessing scripts are provided in the *AQUARIUM-HB* GitHub repository to normalize and adapt the input data. Second, the input RNA-seq data must be rRNA-depleted, a crucial step given the inherently low abundance of circRNAs, as rRNA depletion enhances detection sensitivity, and this requirement is shared by many existing circRNA detection tools. Third, it leverages external long-read datasets for accurate isoform-level reconstruction, which greatly improves structural resolution but may limit the method's performance in tissues or conditions lacking sufficient long-read coverage.

While *AQUARIUM-HB* was validated using bulk blood RNA-seq, its modular framework enables adaptation to cell-free RNA-seq data, a critical area for liquid biopsy. Challenges such as RNA degradation and low input in cell-free samples necessitate preprocessing adjustments like adapter trimming and length normalization. We plan to extend the pipeline to cell-free datasets in future research to enhance its utility for circRNA biomarker discovery in non-invasive diagnostics.

## 5 Conclusion

This study introduces *AQUARIUM-HB*, a comprehensive pipeline capable of identifying and quantifying circRNAs at the transcript level from HT RNA-seq data of human peripheral blood samples. *AQUARIUM-HB* integrates a reference set of full-length blood-derived circRNAs, combining established circRNA databases with new findings from HT RNA-seq datasets to ensure precise circRNA identification and quantification. By applying *AQUARIUM-HB* to a dataset of COVID-19 patients, we demonstrated its potential in uncovering the unique expression dynamics of circRNAs in response to diseases. The pipeline's ability to capture and quantify full-length circRNA structures not only enhances the accuracy of circRNA profiling in blood but also facilitates the exploration of circRNAs as biomarkers in liquid biopsies.

## CRediT authorship contribution statement

**Shaoxun Yuan:** Writing – review & editing, Writing – original draft, Methodology, Formal analysis, Data curation. **Xue Bai:** Methodology. **Linwei Li:** Formal analysis. **Wanjun Gu:** Writing – review & editing, Writing – original draft, Validation, Supervision, Project administration, Investigation, Funding acquisition, Conceptualization.

## Contributions

W.G. conceived the research. S.Y., X.B. and L.L. contributed to data analysis. S.Y. and W.G. wrote the manuscript. All authors read, revised and approved the final version of the manuscript.

## Code availability

*AQUARIUM-HB* is publicly available and can be accessed on GitHub:

Shell-based version: https://github.com/NJUCMbioinfo/AQUARIUM-HB.

R package version: https://github.com/shaoxunyuan/AQUARIUMHB.

## Declaration of competing interest

The authors declare that they have no known competing financial interests or personal relationships that could have appeared to influence the work reported in this paper.
