# Supplementary material for: AQUARIUM_HB: a bioinformatics pipeline for human blood circular RNA analysis
Source: Noncoding RNA Res. 2025 Sep 15;16:32–9. doi: 10.1016/j.ncrna.2025.09.004 (PMC12510082; doi:10.1016/j.ncrna.2025.09.004)
Supplement: Multimedia component 1 [file mmc1.docx]

**Supplementary Materials for Yuan *et al.*, “*AQUARIUM_HB*: a bioinformatics pipeline for human blood circular RNA analysis”**

**Supplementary Table S1.** The number of full-length circRNAs from different tissues in *FLcircAS*^1^ and *IsoCirc*^2^ databases.

|  | **Tissue** | **Full-length circRNAs in *FLcircAS^1^*** | **Full-length circRNAs in *IsoCirc^2^*** |
| --- | --- | --- | --- |
| **Blood** | Blood | 275,165 | 31,998 |
| **Non-Blood** | HEK293 | 478,556 | 56,518 |
|  | Testis | 358,347 | 42,587 |
|  | Brain | 266,890 | 35,762 |
|  | Kidney | 165,697 | 22,496 |
|  | Adrenal gland | 139,912 | 21,953 |
|  | Lung | 115,366 | 18,119 |
|  | Smooth muscle | 81,496 | 16,256 |
|  | Liver | 66,338 | 11,969 |
|  | Heart | 60,181 | 11,210 |
|  | Skeletal muscle | 52,128 | 10,899 |
|  | Adipose | 44,516 | 8,827 |
|  | Prostate | 39,079 | 8,110 |
|  | MCF-7 | 38,589 |  |
|  | VCaP | 36,596 |  |
|  | SH-SY5Y | 31,994 |  |
|  | HEK293T | 23,715 |  |
|  | Cortex | 4,442 |  |
|  | SKOV3 | 64,664 |  |
|  | HeLa | 45,586 |  |

**Supplementary Table S2.** Comparison of *AQUARIUM*-*HB* with published circRNA analysis tools.

| **Tools** | **Library type** | **Analysis module** | **CircRNA level** | **Computational platform** |
| --- | --- | --- | --- | --- |
| *CIRIquant*^3^ | PE, SE | Identification; Annotation;  Quantification | BSJ | Python |
| *CIRCexplorer*^4^ | PE, SE | Identification; Annotation | BSJ | Python |
| *CIRI2*^5^ | PE, SE | Identification;  Quantification | BSJ | Perl |
| *CIRI-full*^6^ | PE | Identification | Isoform | Java |
| *KNIFE*^7^ | PE, SE | Annotation;  Quantification | BSJ | Python, R, Perl, Shell |
| *find_circ*^8^ | SE | Identification;  Quantification | BSJ | Python |
| *CIRI*^9^ | PE, SE | Identification;  Quantification | BSJ | Perl |
| *CIRCexplorer3*^10^ | PE, SE | Identification; Annotation;  Quantification | BSJ | Python |
| *AQUARIUM*^11^ | PE | Identification; Annotation;  Quantification | Isoform | Perl, Java |
| *AQUARIUM-HB* | PE | Identification; Annotation;  Quantification | Isoform | Python, R, Perl, Shell |

PE = paired end; SE = single end; BSJ = back-splicing junction.

**
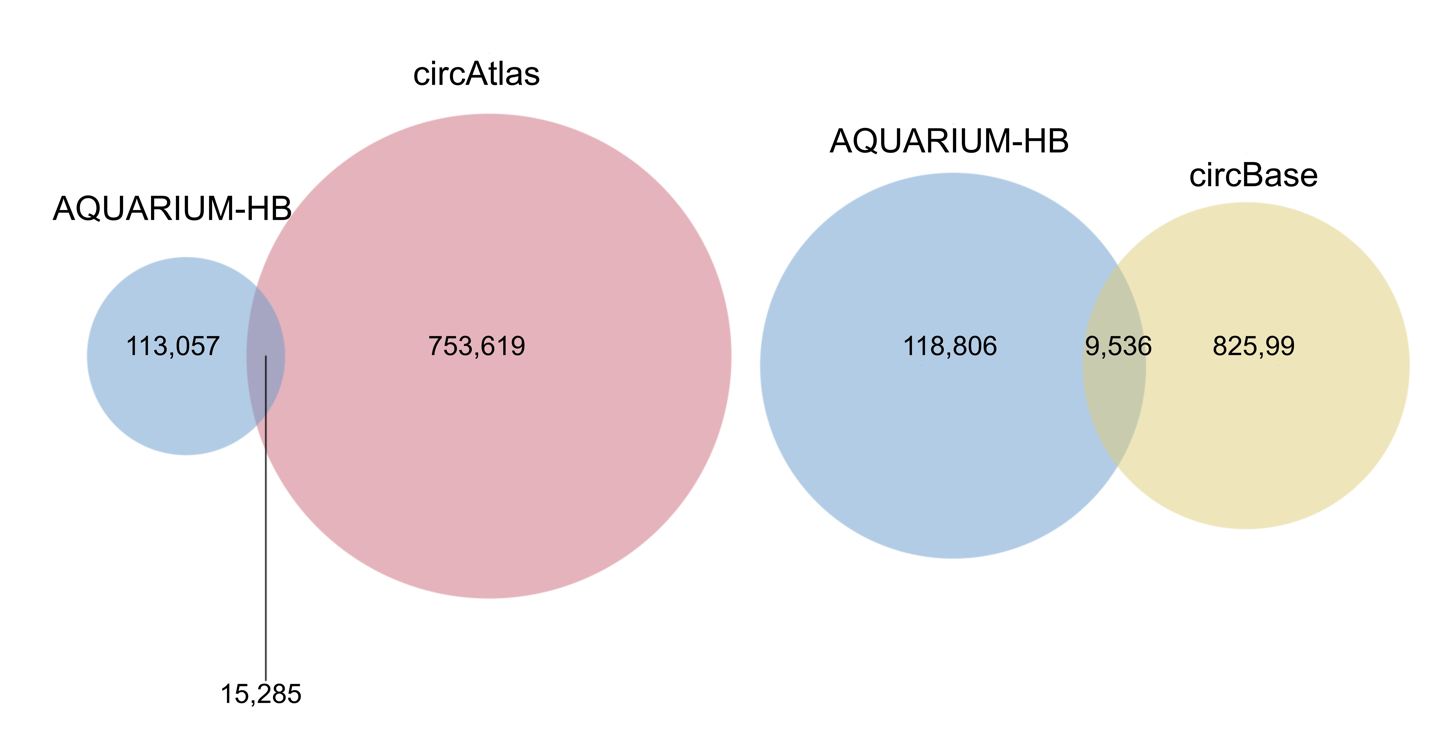
**

**Supplementary Figure S1. The overlap of circRNAs identified by *AQUARIUM-HB* with those recorded in *circAtlas*^23^, and *circBase*^30^.**

**
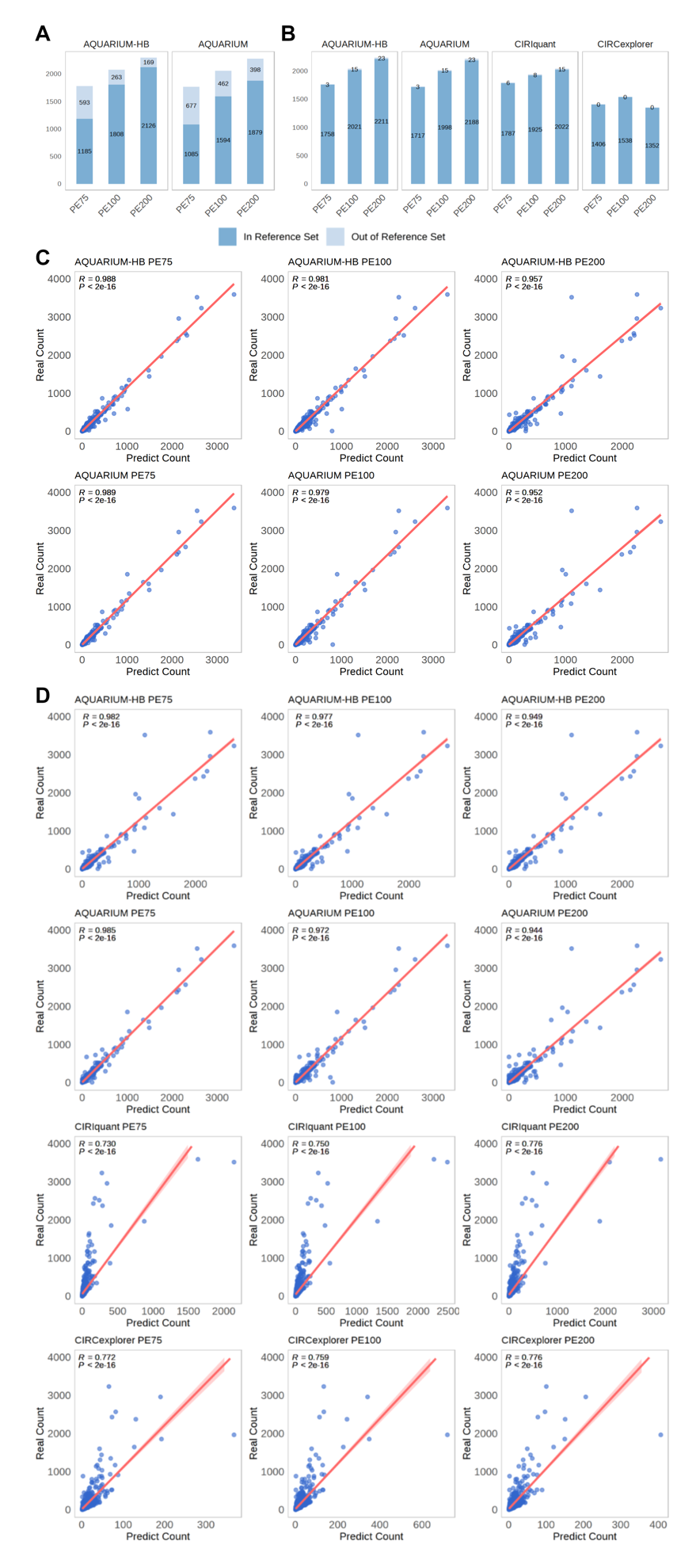
**

**Supplementary Figure S2. Performance comparison of *AQUARIUM*-*HB* with *AQUARIUM*^11^, *CIRIquant*^3^, and *CIRCexplorer*^10^ across three simulated RNA-seq datasets with different read lengths (PE75, PE100, PE200).** **(A)** Numbers of circRNAs detected within and outside the reference set at the isoform level; **(B)** Numbers of circRNAs detected within and outside the reference set at the BSJ level; **(C)** Isoform-level circRNA expression: correlation between tool-detected values (*x*-axis) and simulated ground-truth values (*y*-axis); **(D)** BSJ-level circRNA expression: correlation between tool-detected values (*x*-axis) and simulated ground-truth values (*y*-axis). Pearson correlation coefficients (*R*) and *P*-values are indicated.

**Reference**

1. Chiang, T. W. *et al.* FL-circAS: an integrative resource and analysis for full-length sequences and alternative splicing of circular RNAs with nanopore sequencing. *Nucleic Acids Res.* **52**, D115–D123 (2024).

2. Xin, R. *et al.* isoCirc catalogs full-length circular RNA isoforms in human transcriptomes. *Nat. Commun.* **12**, 266 (2021).

3. Zhang, J., Chen, S., Yang, J. & Zhao, F. Accurate quantification of circular RNAs identifies extensive circular isoform switching events. *Nat. Commun.* **11**, 90 (2020).

4. Ma, X. K., Xue, W., Chen, L. L. & Yang, L. CIRCexplorer pipelines for circRNA annotation and quantification from non-polyadenylated RNA-seq datasets. *Methods* **196**, (2021).

5. Gao, Y., Zhang, J. & Zhao, F. Circular RNA identification based on multiple seed matching. *Brief. Bioinform.* **19**, 803–810 (2018).

6. Zheng, Y., Ji, P., Chen, S., Hou, L. & Zhao, F. Reconstruction of full-length circular RNAs enables isoform-level quantification. *Genome Med.* **11**, 1–20 (2019).

7. Szabo, L. *et al.* Statistically based splicing detection reveals neural enrichment and tissue-specific induction of circular RNA during human fetal development. *Genome Biol.* **17**, 263 (2016).

8. Circular RNAs are a large class of animal RNAs with regulatory potency. *Nature* **495**, 333–338 (2013).

9. Gao, Y., Wang, J. & Zhao, F. CIRI: An efficient and unbiased algorithm for de novo circular RNA identification. *Genome Biol.* **16**, 4 (2015).

10. Ma, X.-K. *et al.* CIRCexplorer3: A CLEAR pipeline for direct comparison of circular and linear RNA expression. *Genomics Proteomics Bioinformatics* **17**, 511–521 (2019).

11. Wen, G. *et al.* AQUARIUM: Accurate quantification of circular isoforms using model-based strategy. *Bioinformatics* **37**, 4879–4881 (2021).

12. Wu, W., Zhao, F. & Zhang, J. circAtlas 3.0: A gateway to 3 million curated vertebrate circular RNAs based on a standardized nomenclature scheme. *Nucleic Acids Res.* **52**, D52–D60 (2024).

13. Glažar, P., Papavasileiou, P. & Rajewsky, N. circBase: A database for circular RNAs. *RNA* **20**, 1666–1670 (2014).
